# Endothelio-hematopoietic relationship: getting closer to the beginnings

**DOI:** 10.1186/1741-7007-9-88

**Published:** 2011-12-28

**Authors:** Sabrina Gordon-Keylock, Alexander Medvinsky

**Affiliations:** 1 Institute for Stem Cell Research, MRC Centre for Regenerative Medicine, 5 Little France Drive, Edinburgh EH16 4UU, Scotland, UK

## Abstract

The close association between hematopoietic and endothelial cells during embryonic development led to the proposal that they may originate from a common ancestor - the hemangioblast. Due to a lack of unique specific markers for *in vivo *cell fate tracking studies, evidence supporting this theory derives mainly from *in vitro *differentiation studies. Teixeira and colleagues describe a novel enhancer that drives specific eGFP expression in blood islands of the electroporated chick embryo, thereby presenting a tool potentially suitable for analysis of hemangioblast differentiation and development of blood islands.

See research article: http://www.biomedcentral.com/1471-213X/11/76

## Commentary

**Figure 1:**
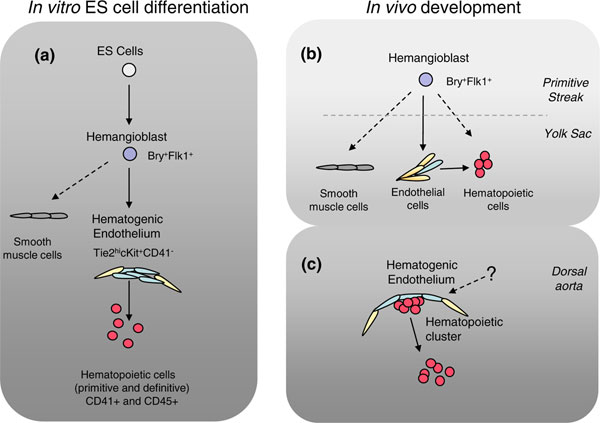
**Modeling hematopoietic development**. **(A) **Hematopoietic differentiation of embryonic stem (ES) cells involves production of mesoderm-derived Brachyury^+^Flk1^+ ^hemangioblasts, which are capable of generating endothelial and hematopoietic cells through an intermediate hematogenic endothelium stage. **(B) **Hemangioblasts have been observed transiently in the primitive streak *in vivo *and their differentiated progeny are thought to colonize the yolk sac. **(C) **At later stages, hematopoietic clusters appear in close association with the hematogenic endothelium within major vessels. Hematopoietic cells bud off and enter the vessel lumen. The question mark indicates that the origin of the hematogenic endothelium of the dorsal aorta in higher vertebrates is not entirely clear.

For over a century researchers have observed an intricate relationship between hematopoietic and vascular endothelial cells. These cell types are closely linked and together they constitute a system that penetrates tissues and organs, thereby providing nutrients, oxygen and immune defense, as well as participating in other important functions. As early as the late 19th century researchers noticed that primitive erythroid and endothelial cells develop in close juxtaposition within structures in the embryonic yolk sac known as blood islands. These subsequently cavitate and remodel to form vascular networks filled with hematopoietic cells (reviewed in [1]). This observation led researchers to postulate the existence of the hemangioblast, a bipotent embryonic precursor cell that can generate both blood and vascular endothelial cells. Other evidence has suggested that hematopoietic cells originate from already specialized endothelial cells, known as hematogenic (hemogenic) endothelium [2]. During midgestation, hematopoietic clusters associated with the endothelial lining of major vessels were found to co-express endothelial and hematopoietic markers, further suggesting a developmental relationship between these two lineages [3]. The current view presents hemangioblasts and hematogenic endothelial cells as two consecutive developmental stages leading to formation of blood [4] (Figure 1A).

Despite a long history of studies in several model organisms, the hemangioblast has remained somewhat elusive. Histological observations and immuno-staining have not allowed direct identification of the hemangioblast in the early embryo due to the rarity of these cells and the lack of known uniquely specific markers; and experimental evidence for the hemangioblast *in vivo *has been obtained only relatively recently using *in vitro *assays [4-7]. How exactly does the hemangioblast behave *in vivo*? In the paper by Teixeira *et al*. [8], the authors describe a green fluorescent protein (GFP) reporter driven by a *cis*-regulatory region of the *Cerberus *gene that labels the earliest cells of blood islands in the chicken yolk sac. Using live fluorescent imaging, the authors observed ingression of the GFP+ cells through the primitive streak - the site of mesoderm and endoderm formation in mammalian, avian and reptilian embryos - during gastrulation, resulting in the formation of yolk sac blood islands. Most hematopoietic cells in yolk sac blood islands and some endothelial cells express the reporter, which led the authors to propose that the initial labeling occurred in the hemangioblast, though further experiments are required to confirm this.

## Studying the hemangioblast: *in vitro *and *in vivo*

What experimental confirmation is there of the existence of the hemangioblast? Due to the relative simplicity of manipulations and the ability to track cell populations of interest, pluripotent embryonic stem (ES) cells became broadly used to model hematopoietic development *in vitro*. The first evidence pointing to the existence of the bipotent hemangioblast was obtained when mouse ES cell-derived progenitors were shown to be capable of producing *in vitro *'blast' colonies (BL-CFC), comprising both hematopoietic and vascular endothelial cells, in response to growth factors [6,7]. ES cell differentiation has been extensively used to model the stepwise transition of the bipotent hemangioblast to the blood and endothelial lineages, shedding light on surface markers expressed at each stage as well as genetic regulation of this process [4,5,8] (Figure 1A). During hematopoietic differentiation, mouse ES cells first generate the primitive streak mesoderm marked by brachyury expression, followed by quick upregulation of Flk1, which marks the formation of extraembryonic mesoderm and the onset of endothelial specification. A fraction of hematogenic endothelial cells subsequently generates CD41+ and CD45+ hematopoietic cells, which critically depends on the transcription factor Runx1. It is broadly accepted that ES cell hematopoietic differentiation replicates yolk sac hematopoiesis; however, both yolk sac and intraembryonic hematopoietic compartments are believed to originate through an endothelial-hematopoietic transition.

Due to limitations in embryonic material and technical approaches, including lack of unique markers, *in vivo *studies of the endothelial-hematopoietic relationship in the early embryo are less abundant. Paradoxically, yolk sac blood islands, which prompted the concept of the hemangioblast in the first place, were for a long time a source of controversy. Initial attempts to identify bipotent cells capable of generating both endothelial and hematopoietic cells failed in both chicken and mouse embryos. Only later was evidence presented that hemangioblasts are transient cells localized to the posterior primitive streak and that by the time of migration to the yolk sac, hematopoietic and endothelial lineages are already segregated [6] (Figure 1B). Interestingly, while in the mouse the blood island appears as a single belt-like structure encompassing the yolk sac [1], blood islands in the chick represent multiple more distinct clusters of cells. One of the most interesting observations made by Teixeira *et al*. using live imaging is that blood island formation in the chick yolk sac involves very dynamic aggregation and recruitment of GFP+ cells during migration from the primitive streak. Thus, despite the morphological appearance of blood islands as individual clones in the chick, their clonal origin seems to be unlikely, as was proposed for the mouse [9]. However, distinct multiple blood islands and availability of genetic markers such as that described by Teixeira *et al*. make the chick embryo a highly attractive model for studying hemangioblast biology and mechanisms underlying the segregation of endothelial and blood lineages.

## Hematogenic endothelium: role in blood production

Although *in vitro *hematopoietic cells can be generated from the hemangioblast through a hematogenic endothelium intermediate, exact details of this process *in vivo *remain obscure. For example, how and where exactly in the embryo do hematopoietic cells transit through an endothelial stage? Do all hematopoietic cells pass through an endothelial stage during embryonic development? In addition, some data indicate that hemangioblasts can generate smooth muscle cells, whereas other reports argue against this [6-10]. The full potency of these cells awaits further elucidation.

The relationship between hematopoietic cells and the endothelium is not limited to yolk sac blood islands. It is broadly thought that the best morphological demonstration of blood cell production by the endothelium is displayed in the midgestational dorsal aorta, umbilical cord and vitelline vessels connecting the embryo proper with the placenta and the yolk sac [11] (Figure 1C). Hematopoietic cells emerging in intra-aortic clusters show distinct morphological features and express markers common to both endothelial and hematopoietic cells, suggesting they are in transition from the underlying endothelium toward a blood cell fate. Individual cells with early hematopoietic markers such as c-kit, CD41 and CD45 can be detected prior to cluster formation in the endothelial layer of the dorsal aorta. Analysis of cultured thick sections of the mouse aorta-gonad-mesonephros region, an area containing intra-aortic hematopietic clusters, have shown that individual cells within the endothelial layer of the dorsal aorta can leave it and migrate inside the aortic lumen [12]. Interestingly, live imaging of zebrafish embryos showed that during an endothelial-hematopoietic transition, cells leave the endothelial lining of the dorsal aorta in the opposite direction to that observed in mouse, and move away from the lumen before entering circulation through the cardinal vein below [13,14].

## Prospective

The importance of finding novel specific markers for hemangioblasts and hematogenic endothelium in higher vertebrates cannot be overestimated. Such specific markers can significantly enhance *in vitro *clonal analysis of endothelial-hematopoietic differentiation and facilitate our understanding of hematopoietic development *in vivo*. Now that a methodology for generating transgenic chickens has become available [15], it is likely that the availability of useful markers and the accessibility of chick embryos for live imaging will significantly increase the use of avian models and will boost research in this area.
